# The reduction of environmentally abundant iron oxides by the methanogen *Methanosarcina barkeri*

**DOI:** 10.3389/fmicb.2023.1197299

**Published:** 2023-07-20

**Authors:** Efrat Eliani-Russak, Zohar Tik, Shaked Uzi-Gavrilov, Michael M. Meijler, Orit Sivan

**Affiliations:** ^1^Department of Earth and Environmental Sciences, Ben-Gurion University of the Negev, Be'er Sheva, Israel; ^2^Department of Chemistry, Ben-Gurion University of the Negev, Be'er Sheva, Israel; ^3^The National Institute for Biotechnology in the Negev, Ben-Gurion University of the Negev, Be'er Sheva, Israel

**Keywords:** methanogenesis, iron oxides, iron reduction, amorphous iron, magnetite, hematite, *Methanosarcina barkeri*

## Abstract

Microbial dissimilatory iron reduction is a fundamental respiratory process that began early in evolution and is performed in diverse habitats including aquatic anoxic sediments. In many of these sediments microbial iron reduction is not only observed in its classical upper zone, but also in the methane production zone, where low-reactive iron oxide minerals are present. Previous studies in aquatic sediments have shown the potential role of the archaeal methanogen Methanosarcinales in this reduction process, and their use of methanophenazines was suggested as an advantage in reducing iron over other iron-reducing bacteria. Here we tested the capability of the methanogenic archaeon *Methanosarcina barkeri* to reduce three naturally abundant iron oxides in the methanogenic zone: the low-reactive iron minerals hematite and magnetite, and the high-reactive amorphous iron oxide. We also examined the potential role of their methanophenazines in promoting the reduction. Pure cultures were grown close to natural conditions existing in the methanogenic zone (under nitrogen atmosphere, N_2_:CO_2_, 80:20), in the presence of these iron oxides and different electron shuttles. Iron reduction by *M. barkeri* was observed in all iron oxide types within 10 days. The reduction during that time was most notable for amorphous iron, then magnetite, and finally hematite. Importantly, the reduction of iron inhibited archaeal methane production. When hematite was added inside cryogenic vials, thereby preventing direct contact with *M. barkeri*, no iron reduction was observed, and methanogenesis was not inhibited. This suggests a potential role of methanophenazines, which are strongly associated with the membrane, in transferring electrons from the cell to the minerals. Indeed, adding dissolved phenazines as electron shuttles to the media with iron oxides increased iron reduction and inhibited methanogenesis almost completely. When *M. barkeri* was incubated with hematite and the phenazines together, there was a change in the amounts (but not the type) of specific metabolites, indicating a difference in the ratio of metabolic pathways. Taken together, the results show the potential role of methanogens in reducing naturally abundant iron minerals in methanogenic sediments under natural energy and substrate limitations and shed new insights into the coupling of microbial iron reduction and the important greenhouse gas methane.

## 1. Introduction

Anoxic environments, such as aquatic sediments, require microorganisms to reduce electron acceptors other than oxygen for their respiration processes. The classical anaerobic respiration order in sediments starts with nitrate reduction (denitrification), with a standard change in free energy (∆G^0^) very similar to aerobic respiration. After nitrate depletion, manganese and iron-oxide reduction occur, followed by sulfate reduction. Ultimately methane production (methanogenesis) becomes the dominant reaction and is considered as the terminal process through which carbon is remineralized in sediments (Froelich et al., 1979).

Of these processes, microbial dissimilatory iron reduction is likely one of the earliest evolutionary metabolic pathways. It plays a vital role in the reductive dissolution of Fe(III) minerals (Weber et al., 2006), the mineralization of organic matter in freshwater sediments (Roden and Wetzel, 2002), and as a redox cycle that drives the biogeochemical cycles of carbon, nitrogen, sulfur, and phosphorus (Kappler et al., 2005; Li et al., 2012). Iron reduction is challenging for microorganisms that depend on low-solubility iron oxide minerals for respiration (Shi et al., 2007), leaving many of these minerals non-reactive for reduction in deep sediments. This leads to the dominance of sulfate-reducing bacteria already in shallow depths, and then methanogenesis by methanogens (archaea). However, significant iron oxide reduction has been observed in marine and fresh water sediments at depths that are much deeper than the classical iron oxide redox zone, at the methanogenic zone, often co-occuring with decrease in methane concentrations (März et al., 2008; Sivan et al., 2011; Slomp et al., 2013; Riedinger et al., 2014; Treude et al., 2014; Bar-Or et al., 2017; Egger et al., 2017; Vigderovich et al., 2019; Amiel et al., 2020).

Reactivation and reduction of low reactive iron oxides in deep methanogenic sediments challenge our understanding of iron reduction, specifically iron-methane couplings and microbial players, with their impacts on the carbon, iron, and other cycles. There are several scenarios and combinations that can explain this reactivation and reductive dissolution of relatively low solubility iron oxides in the methanogenic zone. Among them is a switch of the archaeal methanogens from methanogenesis to iron reduction at the expense of methanogenesis, outcompeting iron-reducing bacteria. The potential role of methanogens from the Methanosarcinales order to reduce iron has been indeed suggested based on geochemical and microbial works on natural sediments (Van Bodegom et al., 2004; Sivan et al., 2016; Bar-Or et al., 2017; Vigderovich et al., 2019; Elul et al., 2021). Previous studies with pure cultures of methanogens showed that it could reduce iron oxides with several external substrates (such as acetate, methanol, or hydrogen; Bond and Lovley, 2002; Liu et al., 2011a; Sivan et al., 2016; Wang et al., 2020). Sivan et al. (2016) also showed that the reduction of amorphous iron by the methanogen *Methanosarcina barkeri* (belonging to the Methanosarcinales order) occurred in a nitrogen atmosphere with the limited substrate in the yeast. Furthermore, incubations of sediments amended with iron-oxides (and acetate as electron donor) have shown an increase in the *Methanosarcina* genus, an indication of the ability of this order to thrive in iron-rich conditions (Gadol et al., 2022).

We hypothesized that these species could also reduce common environmentally abundant less reactive iron-oxide minerals in methanogenic sediments, such as magnetite and hematite, close to the natural conditions (with nitrogen atmosphere), and switch from methanogenesis to iron reduction. This is by oxidizing methane that is highly available there or other organic compounds. Moreover, we speculated that their methanophenazines, or free phenazines that are secreted to their surrounding by other organisms, may promote this reduction.

Phenazines have several important functions in microbes, including population-wide processes such as the regulation of virulence, biofilm formation and related to interspecies signaling (Bassler, 1999; Pierson and Pierson, 2010). As these molecules are highly redox-active, they can also interact with transition metals and mediate important respiratory processes by transferring electrons (Hernandez and Newman, 2001). Unique enzymes that utilize methanophenazines bound to the cell membranes (Abken et al., 1998), mediating the transfer of electrons in the respiratory chain, were found, up to date, only in Methanosarcina species (Mand et al., 2018). The methanophenazines serve as electron transfers in *M. barkeri* in a reactions chain that involves cytochromes, among other proteins (Thauer et al., 2008). Other known electron shuttling molecules, such as quinones or humic substances, may also take part in this process. Humic substances are the high molecular weight products of degradation of plant and microbial matter (Schnitzer, 1978). It was demonstrated in *Methanosarcina acetivorans* that the presence of humic substance analog increases the expression of the MmcA gene, responsible for a *c*-type cytochrome production (Holmes et al., 2019).

Here we investigated the reduction of environmentally abundant iron oxides by pure cultures of *M. barkeri*, and the potential role of electron shuttles in the reduction. This is through a series of short-term (up to two weeks) experiments with various iron oxides and N_2_:CO_2_ atmosphere. We examined the ability of electron shuttles to stimulate the process by adding 9,10-anthraquinone-2,6-disulfonate (AQDS) as an analog to humic substances with known electron shuttling abilities (Lovley et al., 1996). Phenazine-1-carboxylic acid (PCA) was added as an analog to *M. barkeri* methanophenazines function or the free phenazines produced by a variety of bacteria (Thomashow et al., 1990; Hernandez et al., 2004) in many environments, including deep sediments (Thomashow, 2013). The requirement for a direct electron transfer mechanism was explored by adding hematite in vials without direct contact with the *M. barkeri.* As we attempted to mimic the original conditions in methanogenic sediments, we did not focus on the potential electron donors in the media besides methane. We tested whether there is anaerobic oxidation of methane (AOM) in the system by adding labeled ^13^CH_4_ and following its transformation to the dissolved inorganic carbon (DIC) pool.

## 2. Materials and methods

### 2.1. Experimental design

Five short-term experiments (10 to 14 days) were conducted in this study. A pure culture of *M. barkeri* (strain DSM-800 isolated from an anaerobic sewage digester) was obtained from DSMZ (Braunschweig, Germany). Cultures were grown at 37°C and 125 rpm in 110 mL crimp top bottles with rubber stoppers (either Chemglass CLS-4209-14 or GMT stoppers). First, the bottles were prepared with 45 mL of modified phosphate buffer media for methanogens, limited organics (modified after Boone et al., 1989, Supplementary methods S1) and 1% (v/v) vitamin solution (DSMZ medium 141). All media were prepared anaerobically and sterilized under N_2_ purge (all gasses used for purging were of 99.999% grade). To eliminate O_2_ traces, 0.5 mL of titanium-citrate (Ti-Ct) was injected to reach a final concentration of 2.5 mM. Bottles were then purged by H_2_ for 20 min and injected with CO_2_ resulting in a pressure increase of 20% and an H_2_:CO_2_ ratio of 80:20 in the headspace. Media bottles were then inoculated with 2 mL of *M. barkeri* strain DSM-800 at the exponential growth stage. Bottles were kept under the same conditions (37°C and 125 rpm) for approximately two weeks until exponential growth was obtained, as indicated by increasing CH_4_ concentration in the headspace. Then the experiments started by switching to N_2_:CO_2_ (ideally 80:20) atmosphere. Treatments consisted of iron oxides additions in the form of hematite (powder ≥99% <5 μm, Sigma 310,050), magnetite (nano-powder 97%, Sigma 637,106) or poorly crystalline ferrihydrite (amorphous iron; see Supplementary Figure S1 for treatment bottles and SEM images). Amorphous iron was prepared by titrating FeCl_3_ solution with NaOH 10 M until pH 7 was obtained. All additives were prepared with 0.22 μm filtered distilled water and purged with N_2_ for 20 min.

An additional experiment (V) was conducted to ascertain whether *M. barkeri* requires direct contact with the iron oxide in order to reduce it. Hematite was enclosed within predrilled cryogenic vials (2 mL), where the cap was replaced by 0.22 μm PVDF membrane (obtained from Merck Millex® filter unit). The experiment was set up with a 100% N_2_ atmosphere. During the experiment, bottles were kept under 37°C in an incubator and shaken gently each morning (to avoid tearing the cryogenic vials membrane). The full experimental design appears in Supplementary data, Suppelmentary Table S1. Samples were analyzed to determine the concentration of dissolved Fe^2+^, CH_4_, dissolved inorganic carbon (DIC) and δ^13^C of DIC and CH_4_.

Additional control experiments were conducted, comparing treatments without culture (medium was autoclaved and cooled) followed by the addition of vitamins, PCA, and iron oxides (amorphous iron, hematite, or magnetite), as previously described. It should be noted that in all the autoclaved control experiments, iron oxides were added several hours after autoclave, allowing cultures to cool down to ambient temperature to avoid abiotic reactions involving the added iron.

### 2.2. Metabolites extraction

*M. barkeri* cultures containing 10 mM hematite, 100 μM PCA, both of them or none of them (natural cultures) were centrifuged (Allegra™ X-22R Benchtop Centrifuge, Beckman Coulter) for 10 min in maximal RPM in 4°C and filtered with 0.22 μm PVDF filters (Merck Millex®). The filtered supernatant was extracted in solid phase extraction (SPE) method by the manufacture protocol using Biotage® VacMaster™ processing manifold. Briefly, SPE columns (Strata™-X 33 μm Polymeric Reversed Phase 30 mg, phenomenex®) were conditioned and equilibrated with 1 mL MeOH and 1 mL water, respectively. Then, the supernatant was loaded onto the columns; the columns were washed with 1 mL water and dried. The metabolites were eluted from the column with 500 μL 2% formic acid in MeOH twice. The samples were concentrated in vacuum (CentriVap Concentrators, LABCONCO) and kept at-20°C until further analysis.

### 2.3. Mass spectrometry data acquisition

The samples were re-suspended in 200 μL of pre-cooled 1:1 H_2_O:MeOH solution, vortexed, and placed in ice for 15 min. The samples were sonicated for 8 min and centrifuged (Centrifuge 5,424, Eppendorf) for 5 min at 14,680 rpm at room temperature. 150 μL of the supernatant was transferred to mass spectrometry (MS) analysis, and 10 μL were transferred to quality control (QC) vail. The samples were examined by liquid chromatography–tandem mass spectrometry (LC–MS/MS) using an UltiMate 3,000 UHPLC+ focused LC–MS system (Thermo Scientific™) with 2.6 μm Accucore™ C18 (100 × 2.1 mm) HPLC column (Thermo Scientific™), followed by Q Exactive™ Focus Hybrid Quadrupole-Orbitrap™ Mass Spectrometer (Thermo Scientific™) analysis. 5 μL was injected from each sample while the column oven was heated to 40°C. The flow rate was 0.4 mL/min when solvent A was 100% water with 0.1% formic acid, and solvent B was 100% with 0.1% formic acid. The gradients started with 90% of solvent A for 1 min. After 8 min, the gradient was increased to 90% solvent B, and stayed in this composition until 11 min. The gradient changed to 90% A until 12 min, and was maintained that way until 15 min. The scan range was set to 120–1,500 m/z, with a normalized stepped collision energy of 10, 20, 40 eV. The capillary temperature was 275°C, and the spray voltage was 3.5 kV. The MS analysis was conducted in positive ionization modes. To evaluate the instrument operations, the three first and last samples were a test mix sample containing 5-aminofluorescein, caffeine, and theophylline. After the test mix, the QC sample was measured five times, again every six measurements, and five more times in the end. All MS spectra were analyzed by Xcalibur and freestyle software (Thermo Scientific™).

### 2.4. Mass spectrometry data analysis

Acquired MS .raw files were converted to .mzXML files with GNPS (Global Natural Products Social Molecular Networking) Vendor Conversion. The converted files were uploaded to mzMine3 (Pluskal et al., 2010) for feature detection and feature alignment. MzMine3 output was uploaded to GNPS and Sirius for further analysis. To learn more about the relationships and structures of the metabolites in the cultures, a molecular network was created in GNPS (Wang et al., 2016; Schmid et al., 2021) with precursor ion mass tolerance and fragment ion mass tolerance of 0.02 Da, and cosine score of 0.7 to form a network edge. An analysis with MolNetEnhancer followed the molecular networking (Ernst et al., 2019) to assess the chemical family classification of the samples’ metabolites. We analyzed the results based on the direct parent MolNetEnhancer classification. The Sirius tool (Dührkop et al., 2015, 2019; Djoumbou Feunang et al., 2016; Kim et al., 2021), with CSI:FingerID and CANOPUS, was used to annotate a putative identification of the compounds’ structure. MetaboAnalyst (Pang et al., 2022) was used to create principal component analysis (PCA) based on the MS data.

### 2.5. Other analytical methods

Samples for Fe^2+^ and DIC were prefiltered (0.22 μm). One milliliter was immediately fixed using the Ferrozine method (Stookey, 1970) for Fe^2+^ measurement. Ferrous iron absorbance after fixation was measured on a spectrophotometer (562 nm) with a detection limit of 1 μM. Additional 1 mL samples were kept to determine DIC concentrations and δ^13^C_DIC_ analyses at 4°C for up to 1 week prior to measurement. The δ^13^C_DIC_ was measured on a continuous flow Isotopic Ratio Mass Spectrometer (IRMS, DeltaV, Thermo), interfaced to Gas Bench II (GBII), and is reported in per mille units relative to Vienna Pee Dee Belemnite (VPDB) with a precision of 0.2 ‰. DIC was measured on the same IRMS using the peak area with a precision of 0.1 mM. Methane and ${}^{13}C_{{\mathrm{CH}}_{4}}$ were measured from the headspace. Concentrations were measured on a Gas Chromatograph (Focus GC, Thermo) equipped with a flame ionization detector (FID) and packed column (Shincarbon ST) with a detection limit of 1 nmol. The ${}^{13}C_{{\mathrm{CH}}_{4}}$ was measured on the same IRMS with PreCon device addition to GBII.

Gene copies of 16S rRNA were measured by sampling 10 mL culture from experiment IV bottles on day 11. Samples were centrifugated at 9,000 g for 10 min and the pellet was used for DNA extraction (DNeasy PowerLyzer PowerSoil Kit, QIAGEN). DNA concentrations in samples were evaluated using a nanodrop spectrophotometer (ND1000). Real-time PCR amplifications were conducted using an Azure Cielo 3 system (Azure biosystems, United States) in 20 μL volumes, with each standard reaction mix containing 10 μL of SYBR™ Green PCR Master Mix (Applied Biosystems™), 0.8 μL of each primer (F341 and R518) (Muyzer et al., 1993), 6.4 μL distilled water and 2 μL of either DNA or standard (plasmid) as a template. Thermocycling was as follows: 95°C for 5 min, 30 cycles of 10 s at 95°C, 20 s at 62°C, 30 s at 72°C, 2 s at 78°C. Runs were completed with a melting analysis (65°C to 95°C, ramp, 0.5°C/min) to check for product specificity and primer dimer formation. The assay included a standard curve of six standards (from 2.5·10^8^ to 2.5·10^3^ copies per μL) that were freshly prepared from a stock solution, with an efficiency of 101.5 and an R^2^ = 0.996. All standards were run in duplicate and samples in triplicates.

## 3. Results

**Figure 1:**
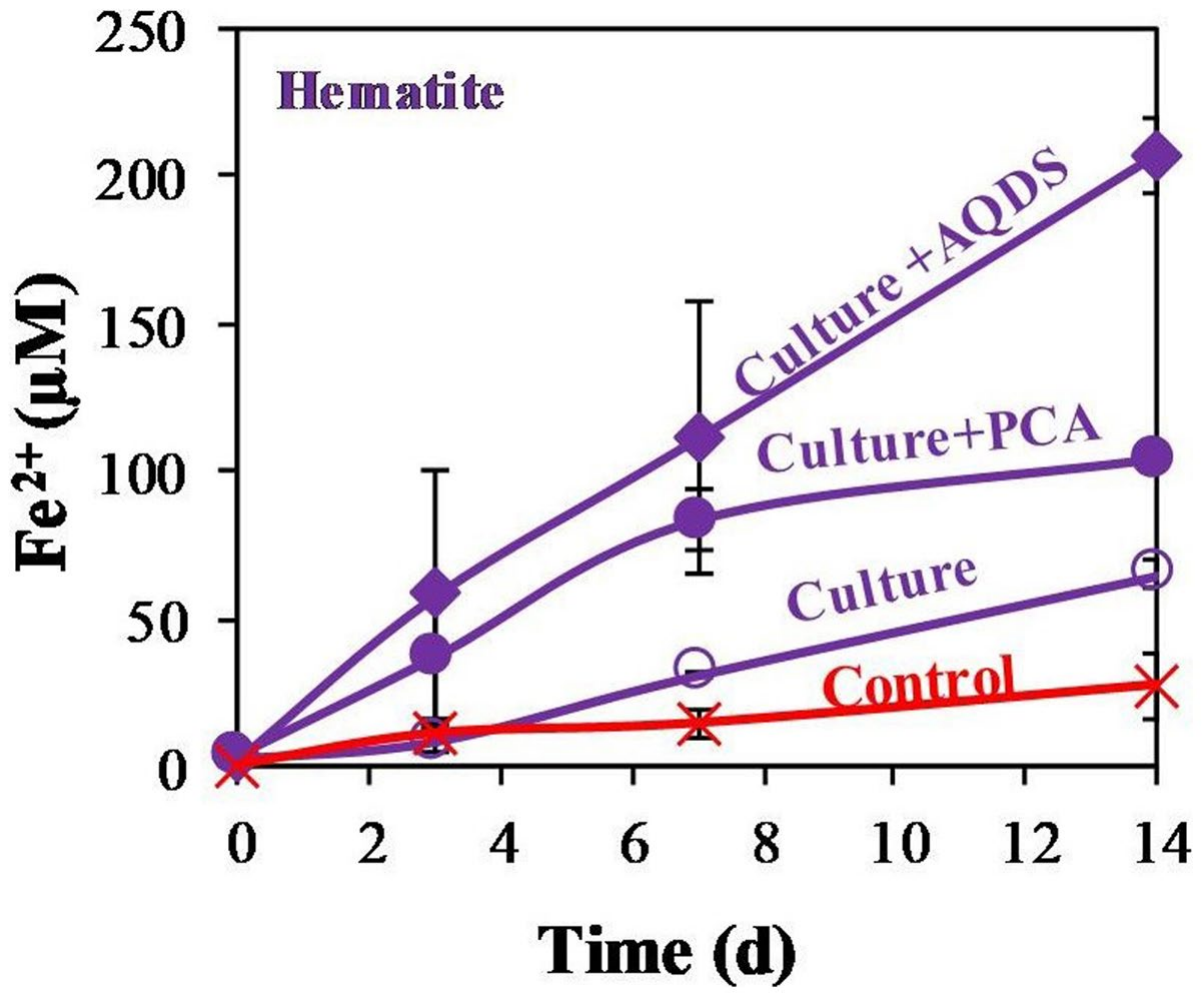
Experiment I: Fe^2+^ production by *M. barkeri* with 1 mL 99% ^13^CH_4_, 10 mM of hematite (so called culture), and 0.1 mM electron shuttles treatments (AQDS or PCA). Control treatment refers to killed culture with AQDS. Error bars represent the standard deviation of triplicates and are not shown when smaller than the symbol.

This study quantified the reduction of abundant low reactive iron minerals found in methanogenic aquatic sediments by methanogens under limited energy, and the effect of this reduction on the efficiency of methanogenesis. For this purpose, we tested the ability of *M. barkeri* to reduce a range of iron oxides, including amorphous iron and the less reactive iron oxides magnetite and hematite. Incubation experiments were conducted using N_2_:CO_2_ headspace without external substrate, besides the organics in the yeast (and methane in one experiment) and both with and without various compounds that are known to function as efficient electron shuttles. Additional separate control experiment containing media control and dead culture control with PCA and amorphous iron, hematite, or magnetite showed little abiotic iron reduction (data not shown), significantly lower than the treatments with the living cultures. In the first experiment (Figure 1), *M. barkeri* was grown anaerobically under N_2_:CO_2_ (90,10) and 1 mL labeled ^13^CH_4_ (99%) atmosphere. The treatments consisted of additions of hematite (10 mM), with or without PCA (0.1 mM), or AQDS (0.1 mM). The results show clearly that *M. barkeri* can reduce hematite, even without the addition of electron shuttles. During the 14 days of the experiment, concentrations of Fe^2+^, the product of iron reduction, increased by about 50 μM upon hematite addition, while combined hematite and PCA addition showed a higher increase in Fe^2+^ production. The largest increase was observed upon the combined addition of hematite and AQDS (0.1 mM), increasing Fe^2+^ concentrations by a factor of 3 to 4 compared to hematite treatment. The δ^13^C_DIC_ remained close to about-30‰ throughout the experiment (data not shown), indicating that no labeled ^13^C-CH_4_ was transformed to ^13^C-DIC, and thus no AOM appeared to occur over this time scale.

**Figure 2:**
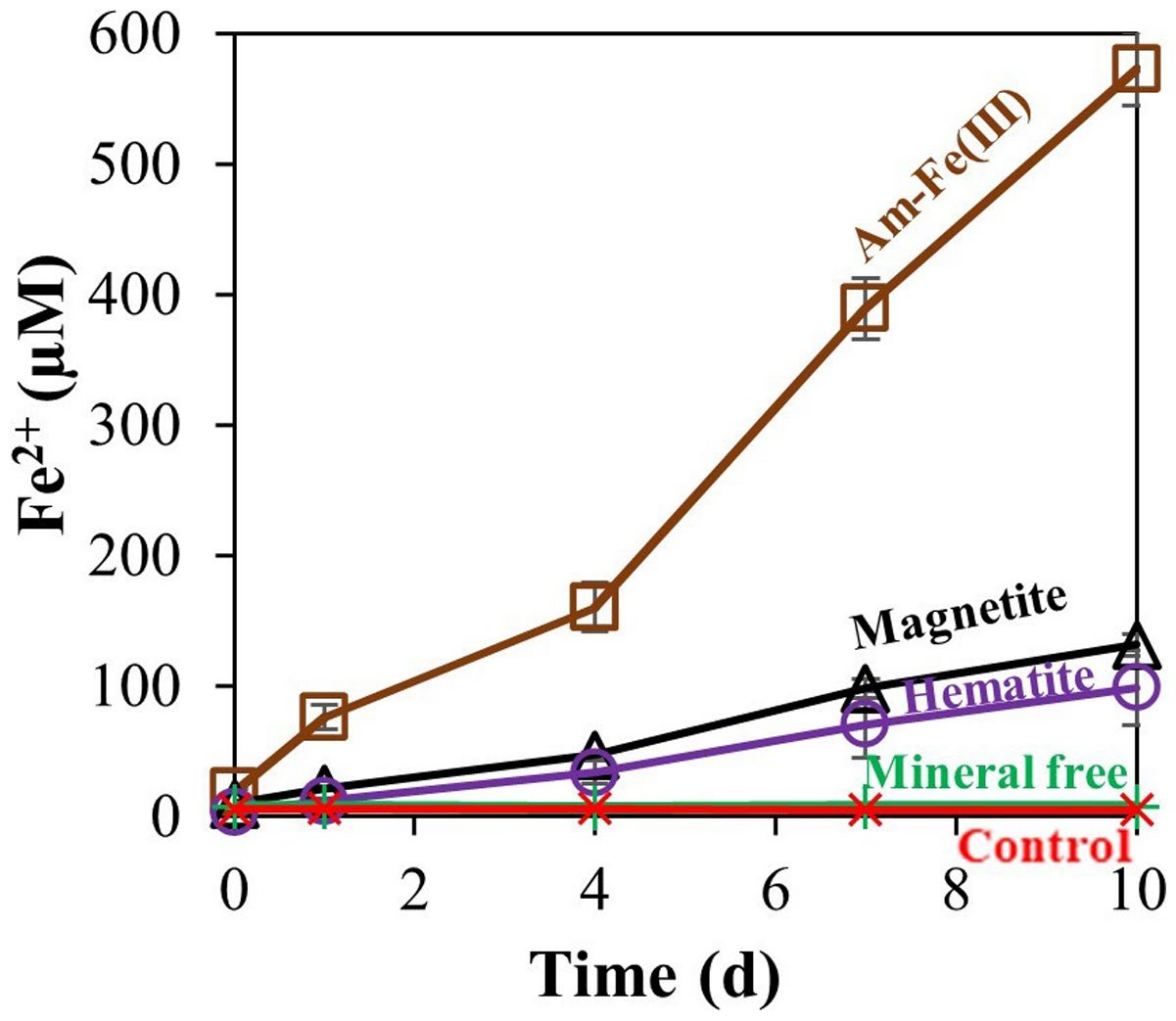
Experiment II: Fe^2+^ production by *M. barkeri* with 10 mM of different iron oxides (amorphous iron, hematite, or magnetite). Treatment “mineral free” refers to the absence of iron oxides, and control refers to killed culture*.* Error bars represent the standard deviation of triplicates for the oxides treatments and are not shown when smaller than the symbol.

After observing that *M. barkeri* can reduce the low reactivity hematite, we proceeded to a second experiment, addressing the reactivity order of different iron oxides (Figure 2). Amorphous iron, magnetite, and hematite were added to *M. barkeri* cultures at a final concentration of 10 mM. On the last day of the experiment (day 10), no iron was produced in the mineral free and killed treatments. Hematite addition resulted in the lowest amount of reduced Fe^2+^ accumulation (1.0 ± 0.3%), while magnetite reduction (1.32 ± 0.09%) resulted in slightly (yet significantly) higher Fe^2+^ concentrations than hematite reduction. Amorphous iron addition resulted in the highest Fe^2+^ concentration increase (5.7 ± 0.3%).

**Figure 3:**
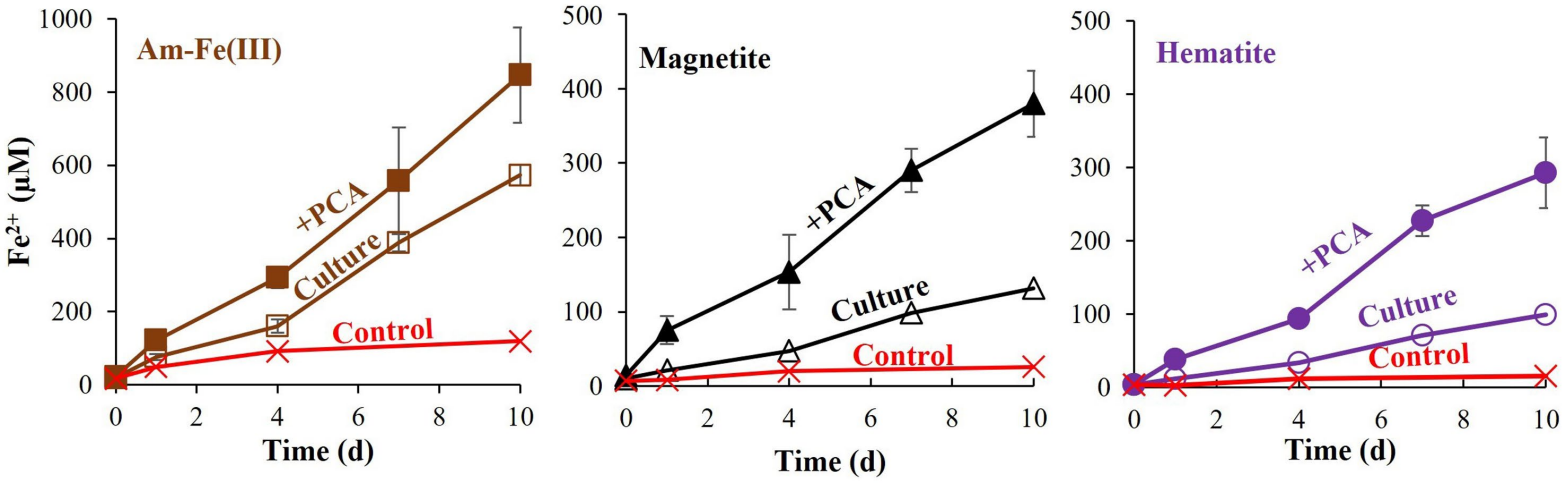
Experiment II: Fe^2+^ production by *M. barkeri* with 10 mM of different iron oxides (amorphous iron, hematite, or magnetite) with and without 0.1 mM PCA. Each iron oxide treatment contained control (killed culture+ iron oxide+ PCA), Culture (culture with iron oxide) and” + PCA” (containing culture, iron oxide, and PCA). Error bars represent the standard deviation of triplicates and are not shown when smaller than the symbol.

**Figure 4:**
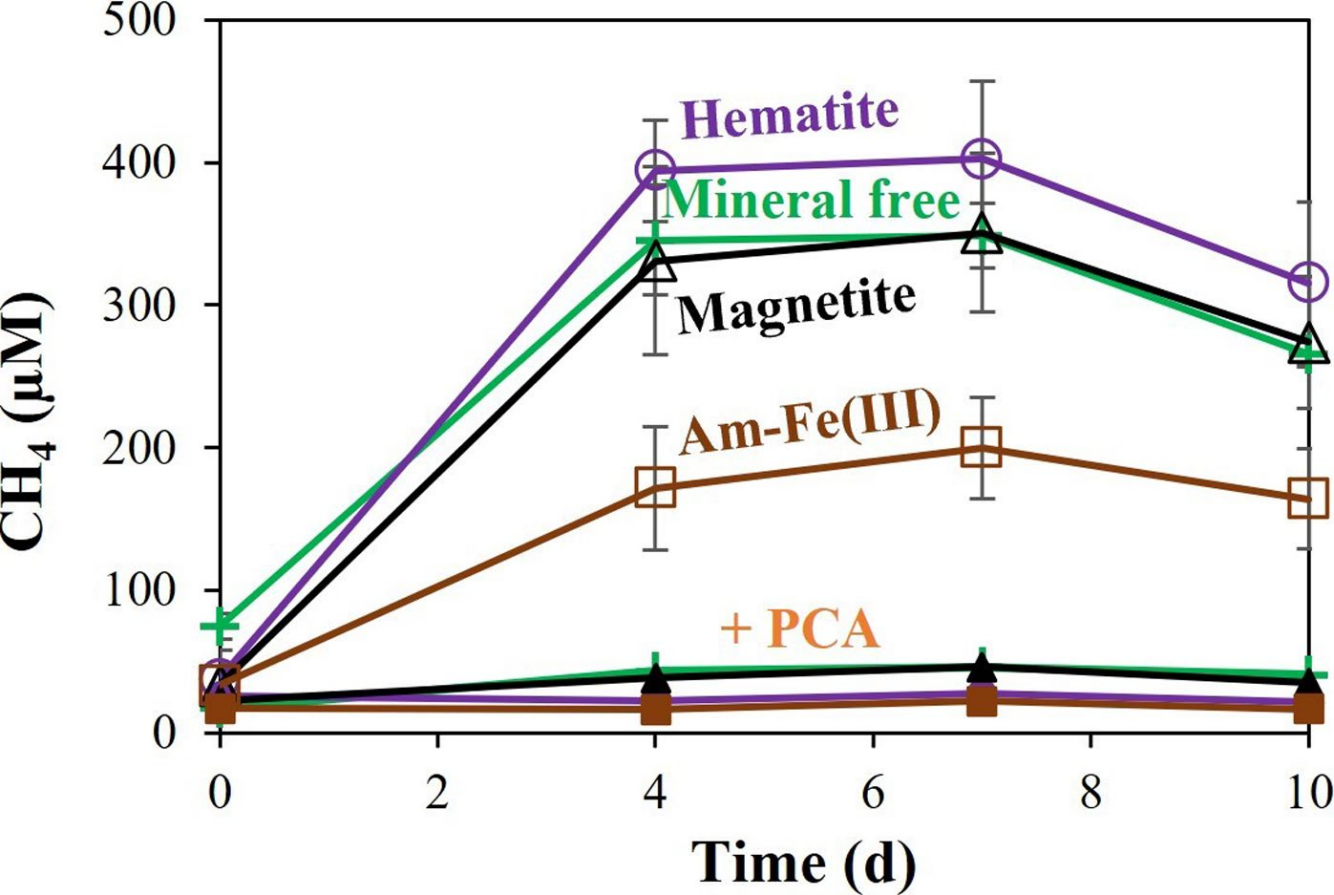
Experiment II: Methane production by *M. barkeri* with 10 mM of different iron oxides (amorphous iron, hematite, or magnetite) with and without 0.1 mM PCA. “+PCA” stands for the treatments with PCA. Error bars represent the standard deviation of triplicates and are not shown when smaller than the symbol.

Additional treatments addressed the effects of PCA addition on the reduction of each iron oxide (Figure 3). In all cases, PCA increased iron reduction compared to treatments without PCA. As was shown for the hematite, the relative increase was the lowest with the hematite treatment, slightly higher for the magnetite and highest for the amorphous iron. Methane was measured in significant quantities in all non-killed bottles without PCA. However, its production stopped before day 4 (Figure 4), while iron reduction continued to increase. In the presence of PCA, methanogenesis was almost completely inhibited from the beginning. In this experiment, we traced the natural changes in ${}^{13}C_{{\mathrm{CH}}_{4}}$ due to carbon isotopic fractionation. These values at the end of the experiment were-38.6 ± 2.2 ‰ (n = 8) for the bottles that produced methane, while with the PCA additions, ${}^{13}C_{{\mathrm{CH}}_{4}}$ value was-49.0 ± 1.1 ‰ (n = 8), fitting lower production of CH_4_.

**Figure 5:**
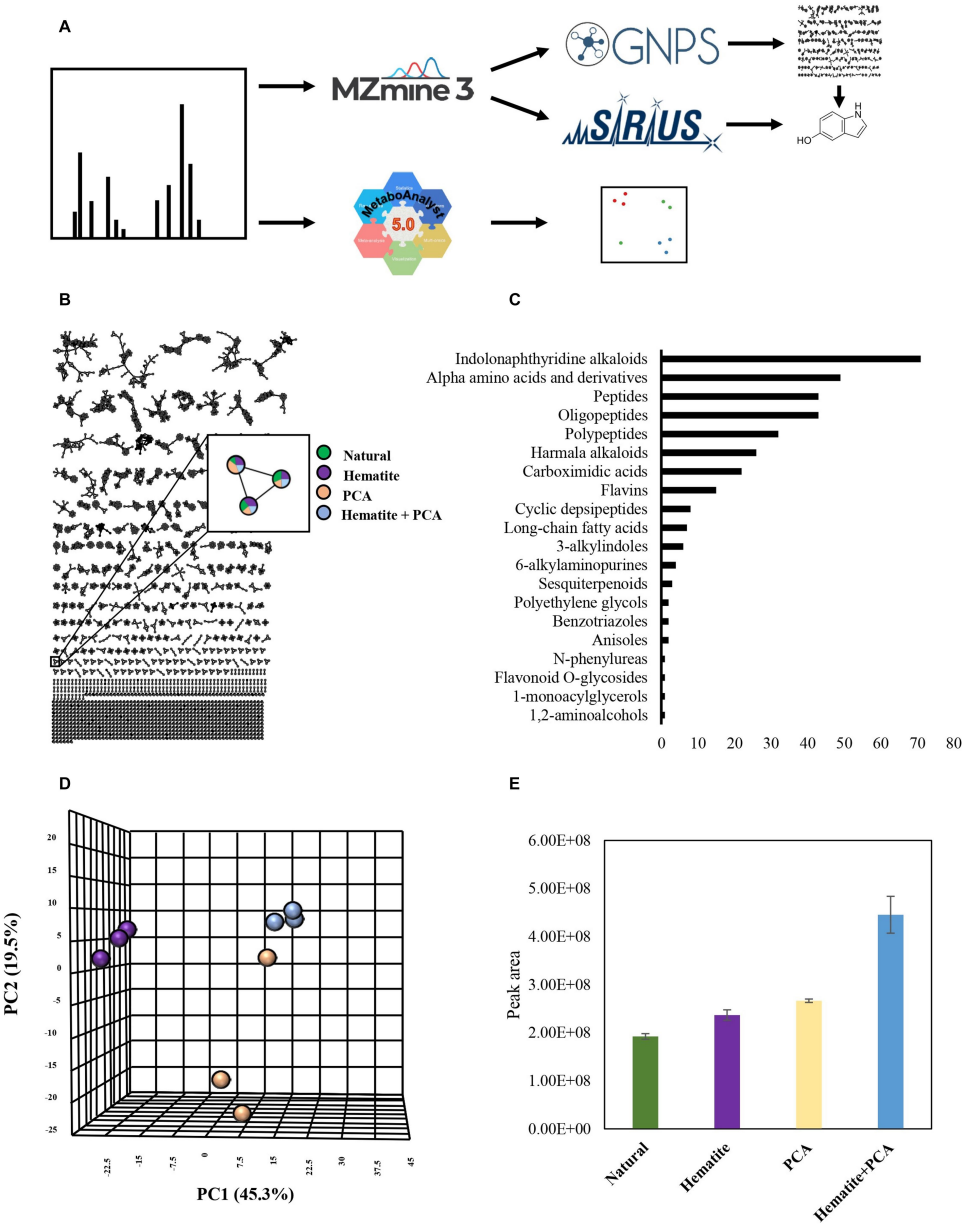
Experiment III: **(A)** Experimental workflow. MS data of cultures containing 10 mM hematite, 0.1 mM PCA, both of them (hematite + PCA) or none of them (natural), was submitted to MzMine3 for peak detection and alignment. The output was uploaded to GNPS and Sirius to create a molecular network and obtain putative structural annotations. The raw data was also submitted to MetaboAnalyst for generating PCA plots. **(B)** Molecular networking created by GNPS. Green-natural, purple-hematite, beige-PCA, blue-Hematite+PCA. Each node represents a metabolite in the sample, and any edge connecting two nodes suggests that they have a similar structure. **(C)** The number of metabolites that were identified as belonging to a chemical family based on MolNetEnhancer results. **(D)** PCA results are based on the samples’ MS data. **(E)** The average peak area of metabolite with m/z of 134.0599 in the different treatments. Error bars represent the standard deviation of triplicates. **(D)** + **(E)** The color legend is the same as **(B)**.

Moreover, in experiment III, we examined *M. barkeri* metabolite production differences in natural cultures and cultures that contained PCA, hematite, or both combined. To identify metabolite production and specific metabolites in the different samples, we generated a molecular network using the GNPS platform (Wang et al., 2016; Figures 5A,B). GNPS clusters together similar structural compounds and compares the metabolites to its database in order to obtain structural information. Based on the molecular networking results, we analyzed the data with the MolNetEnhancer tool, which enables us to predict metabolite family chemical classifications (Ernst et al., 2019). Figure 5C represents the metabolite families that were detected by MolNetEnhancer. The majority of the metabolites, as can be expected from a living organism, are related to amino acids and peptides. The second compound family is based on indoles, which are involved in various known activities in microbes, including in quorum sensing (Lee et al., 2015). There are notable differences between all the experimental treatments, as shown in the PCA plot generated using the MetaboAnalyst platform (Pang et al., 2022; Figure 5D).

The most notable difference was observed for the hematite-containing cultures. Interestingly, the cultures that contained both hematite and PCA did not contain any unique metabolite; however, for several metabolites changes in their production and consumption levels were observed between the different treatments. We examined those metabolites for putative identification by GNPS (Wang et al., 2016) and Sirius algorithms. One of those metabolites has a molecular mass of 134.0599 m/z (positive ionization mode; Figure 5E) and was identified by Sirius as 5-hydroxyindole. Although we were able to refute this annotation by comparing it to a commercial standard with different retention times, the MS2 fragmentations have similar m/z values (even though with different intensities), indicating that this compound probably has a similar structure to hydroxyindole.

**Figure 6:**
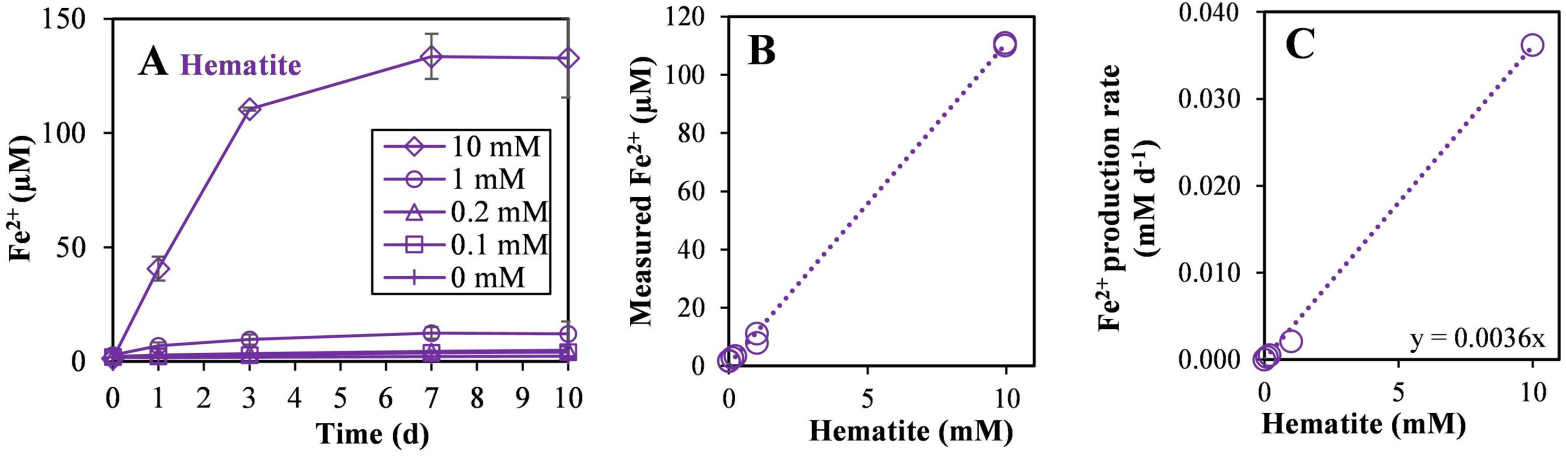
Experiment IV: **(A)** Fe^2+^ production with different hematite concentrations (0, 0.1, 0.2, 1, and 10 mM) during the 10 days of the experiment, **(B)** Fe^2+^ concentration on the third day of the experiment versus the added hematite concentration and, **(C)** iron reduction rate in the first 3 days versus the added hematite concentration. The slope of the linear curve is marked (*n* = 8).

In the fourth experiment, the rate of hematite reduction and the reaction order were quantified. Hematite was added at several concentrations (0, 0.1, 0.2, 1.0, 10 mM). During the experiment, the strain reduced about 1% of introduced hematite in all non-killed incubations (Figure 6A). The linear ratio between the produced Fe^2+^ to added hematite (Figure 6B) suggests that the reaction is of first order, depending solely on hematite concentration. To calculate the reaction coefficient rate, we plotted the production rate of Fe^2+^ during the first three days of the experiment against the concentrations of hematite addition in each culture (Figure 6C). Referring to iron-reduction by *M. barkeri* as a first-order reaction, we calculated the reaction rate based on the plot in figure 6C, leading to the following first-order reaction equation (equation 1), where k is the reaction rate coefficient:

(1) $$\;{\left[Fe^{2+}\right]}_{t}={\left[FeIII\right]}_{0}.\left(1-e^{-kt}\right)$$

Assuming no significant change in the concentration of the hematite with time, the calculated reaction coefficient rate on day 3 of the experiment was found to be 3.60 × 10^−3^ day^−1^. The addition of iron oxides and iron reduction resulted in a lower production of methane, as can be seen by plotting methanogenesis versus iron reduction rates (Supplementary Figure S2). This decrease in methanogenesis was very strong upon the addition of PCA.

**Figure 7:**
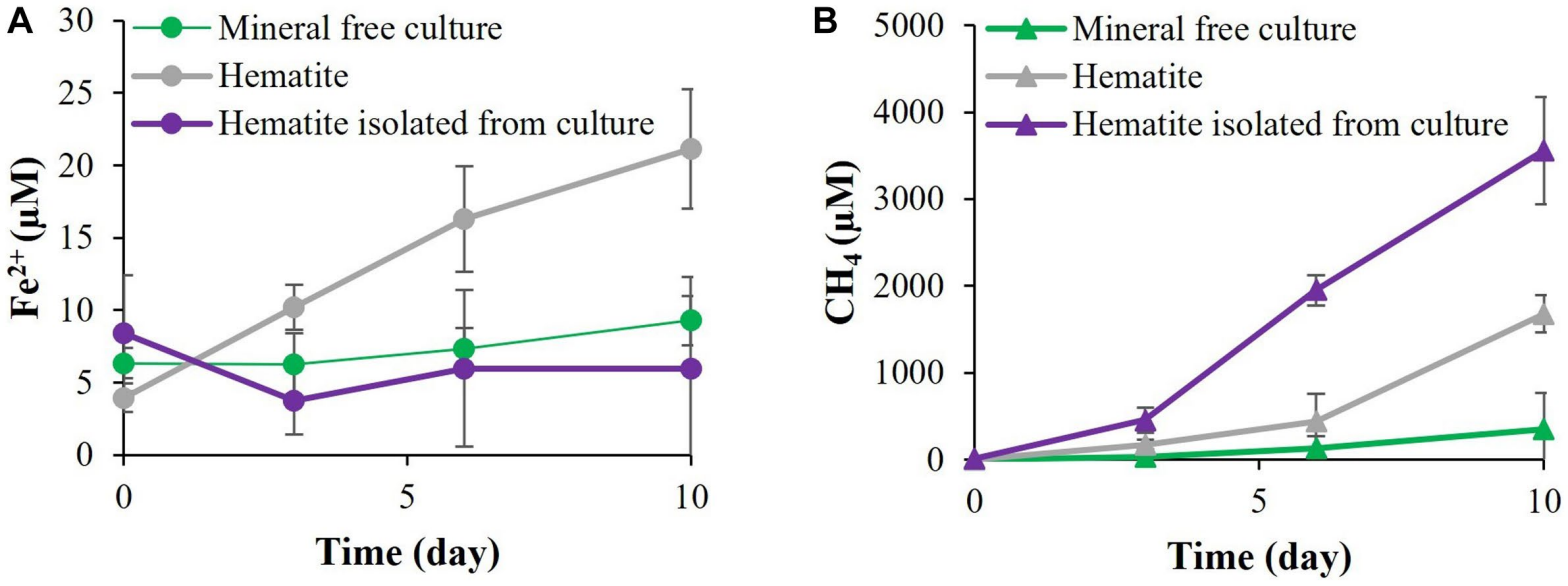
Experiment V: **(A)** Fe^2+^ production with *M. barkeri* allowing direct contact with 10 mM hematite or prevented contact by inclosing the hematite in 0.22 μm capped vial. **(B)** Methane concentrations at the above conditions.

In the fifth experiment, the requirement for direct contact with the iron minerals was tested by adding hematite inside vials without direct contact with *M. barkeri* (Figure 7). When the hematite was added without direct contact to the *M. barkeri*, no iron reduction was observed, and methanogenesis was not inhibited, as compared to the addition hematite directly to the media. As expected, enhanced methanogenesis (in the treatment with hematite inside the vials) was accompanied by a slight increase in the DIC and enriched carbon isotopic values, compared to the treatment without the vials (data not shown).

## 4. Discussion

In many natural aquatic sediments, intensive iron reduction by the natural abundant iron mineral appears well below its classical region, below the sulfidic zone in the deep methanogenic zone, leading to accumulation of dissolved Fe^2+^ (März et al., 2008; Sivan et al., 2011; Slomp et al., 2013; Riedinger et al., 2014; Treude et al., 2014; Bar-Or et al., 2017; Egger et al., 2017). Theoretically, four scenarios and their combinations can explain iron reduction in the methanogenic zone: (i) Methane, as a dissolved gas, may be more available there than other organic substances, which may be already refractory. That will drive microbial activity toward AOM with iron as an electron acceptor (Bar-Or et al., 2017; Cai et al., 2018); (ii) There are cryptic redox cycles between iron-oxides and species that are abundant in the methanogenic zone. This may include reactions with sulfide minerals (Fike et al., 2015) or with ammonium (Clément et al., 2005; Sawayama, 2006; Yang et al., 2012; Ding et al., 2014; Li et al., 2018; Yao et al., 2019); (iii) Iron reducing bacteria outcompete methanogens on common substrates (Roden, 2003; Lovley, 2006) and (iv) Methanogens may switch from methanogenesis to iron reduction at the expense of methanogenesis and outcompete iron-reducing bacteria (Bond and Lovley, 2002; Van Bodegom et al., 2004; Sivan et al., 2016).

This study tested the plausibility and the benefit of the last scenario to occur by quantifying the ability of pure methanogens to reduce environmentally abundant iron minerals close to natural conditions and examining their effect on methanogenesis. Several studies have already shown that methanogens are able to use iron oxides as electron acceptors. This was examined with amorphous iron (Bond and Lovley, 2002; Van Bodegom et al., 2004; Sivan et al., 2016), clay minerals (Liu et al., 2011a; Zhang et al., 2012, 2013), goethite (Liu et al., 2011b) or magnetite (Wang et al., 2020). However, the reduction at close to natural conditions, without the addition of external electron donor as hydrogen acetate or methanol, was tested only for amorphous iron and some types of clays (smectite and illite/smectite). Experiments on natural sediments with the addition of iron-oxides have been shown increases in the *Methanosarcina* order (Gadol et al., 2022), which *M. barkeri* belongs to. Here we attempted to mimic the natural conditions by exploring the archaeon methanogen *M. barkeri* and its switch between methanogenesis and iron reduction using an atmosphere of N_2_:CO_2_ (80,20) and abundant iron oxide minerals. We also explored the reduction mechanism with an emphasis on the potential advantage of Methanosarcinales in reducing iron over iron-reducing bacteria thanks to the methanophenazines found on their membrane with known electron shuttling abilities. The oxides were those found in methanogenic marine and lake sediments [e.g., ref. (Bar-Or et al., 2017; Vigderovich et al., 2019; Amiel et al., 2020)] and included amorphous iron, magnetite, and hematite. Magnetite and hematite have very low solubility (Lovley, 2006) and are considered to be the least reactive minerals for reduction (Canfield, 1989; Roden and Zachara, 1996; Poulton et al., 2004). Here we discuss our main findings.

### 4.1. *Methanosarcina barkeri* can utilize a variety of iron-oxides

*Methanosarcina barkeri* is known as a hydrogenotrophic methanogen, which can also use acetate and methanol as substrates (Bond and Lovley, 2002). Our results show that *M. barkeri* is also able to use other metabolism and transfer electrons to iron minerals. It can reduce several iron-oxide minerals at close to natural conditions, with the organic substrate in the medium (Figures 1, 2). The concentrations of the most abundant iron oxides can vary widely depending on the settings. The concentration used in the study were similar to those found in natural sediments (Canfield, 1989; Bar-Or et al., 2017; Boyko et al., 2021). As was anticipated by other studies (Munch and Ottow, 1983), amorphous iron was the most reactive iron-oxide in our experiments (Figure 2). Magnetite and hematite showed much lower reactivity, where more iron was reduced in the presence of magnetite, leaving hematite as the least reactive metal oxide in this experiment (Figure 2). In the killed cultures and media control experiments the amount of reduced iron was negligible. The question is how does *M. barkeri* overcome the kinetic barrier that is usually involved in the reduction of low-solubility iron minerals?

### 4.2. Promotion of iron reduction by *Methanosarcina barkeri* by electron shuttles

Microbial reduction of iron oxides is a challenging process. The environmentally abundant iron-oxides are insoluble (Kappler and Straub, 2005), and the cell membrane forms a highly impermeable barrier (Shi et al., 2007). Microorganisms, however, have developed several mechanisms to overcome this challenge: (i) Electron carriers from the *c*-type cytochrome family are used to transfer electrons from the inner side of the membrane to the cell surface, and eventually to the mineral (Mehta et al., 2005); (ii) extension of the surface that can take part in this process by conductive pili or nanowires enable the electron transfer from the microbe to the mineral (Gralnick and Newman, 2007); (iii) secretion of iron chelators (siderophores) that can chelate Fe(III) tightly, convert this relatively insoluble iron to a bioavailable substance, which can then be utilized by the cell (Lippard and Berg, 1994), and (iv) reduction of electron shuttles that are subsequently oxidized by mineral iron oxides, effectively recycling the electron shuttles.

Several known compounds can participate in the last mechanism, e.g., cysteine (Doong and Schink, 2002), humic substances, and various redox-active compounds that can be produced by microbes, such as phenazines (Hernandez et al., 2004). A major class of redox molecules used as electron shuttles is the class of quinones. Quinones can be found in all living domains (Nowicka and Kruk, 2010). However, in methanogens they are usually found only in very low amounts (Hughes and Tove, 1982; Abken et al., 1998). Microbes, however, also often use redox compounds that are not produced by themselves, explaining *M. barkeri’s* ability to utilize both humic acids and the related quinone analog AQDS (Bond and Lovley, 2002).

*M. barkeri* has been shown to use these electron shuttles to increase the availability of Fe(III) in amorphous iron oxides (Roden and Zachara, 1996; Scott et al., 1998). Here, we confirm that *M. barkeri* can exploit AQDS to reduce not only a high-reactivity iron species such as amorphous iron, but also less reactive iron minerals (Figures 1, 2) and under close to natural conditions (N_2_:CO_2_ atmosphere). The reaction between the AQDS and available iron species is an abiotic process. Some iron reduction was observed in control cultures containing dead archaea. Nevertheless, in the absence of reducing organisms the AQDS cannot be recycled, limiting the amount of reduced iron. The archaea reduce more iron than the killed control in the presence of only hematite (without AQDS), validating the crucial role that the living microbes play in the iron-reduction process. When AQDS was added to live cultures containing hematite, the iron reduction increased dramatically, supporting the assumption that this process can indeed occur in nature.

Although methanogens do not produce quinones, they possess other membrane-bound redox electron carriers, such as methanophenazines (Abken et al., 1998; Beifuss et al., 2000). It is possible that electron shuttling by methanophenazines can be utilized for additional energy generation processes, such as iron reduction. Methanophenazines and cytochromes were recently found to be also involved in ferrihydrite-dependent AOM (Yan et al., 2018; Yu et al., 2022), but AOM did not seem to occur in our cultures. Phenazines are known to promote mineral iron reduction (Hernandez et al., 2004) thanks to their favorable redox potential. Due to their lipid character, methanophenazines are highly insoluble in aqueous environments (such as the culture media). Therefore, in order to examine their potential more soluble analogs such as PCA are added (Abken et al., 1998). PCA was also added to mimic free phenazines that are abundant in sediments (Thomashow, 2013). The addition of PCA enhanced iron reduction in all samples (Figures 1, 3). It did not affect the reactivity order of the different minerals, i.e., more iron-reduction occurred in the presence of amorphous iron and PCA, while the lowest amount was observed when hematite was added to PCA containing cultures. Some iron reduction was observed in the killed control cultures due to abiotic reactions, emphasizing the crucial role of microorganisms in cycling the electron shuttle, thus enabling the process to continue.

Moreover, the addition of PCA to the media with the iron mineral released more metabolites than without it, but no unique metabolites were detected in cultures containing both hematite and PCA. This change in the metabolite levels, but not in their identity, suggests that the combination of the iron mineral and the electron shuttle enhances existing pathways. This observation supports the possibility that *M. barkeri’s* own phenazines participate in the iron-reduction. As methanophenazines are strongly associated with the microbial membranes, their potential role in iron reduction by *M. barkeri* versus free phenazines was supported by the experiments where hematite was added to the media directly or inside a vail, which enabled only the transfer of dissolved species. The results indicate clearly that direct contact between the mineral and the cells is needed for electron transfer.

One of the major metabolite groups that was found is the indole family. Indoles are known for their role in biological processes such as signaling (Lee and Lee, 2010; Tomberlin et al., 2017) and regulation (Hu et al., 2010; Duca and Glick, 2020), and can strongly affect the growth and behavior of organisms from all kingdoms (Bennett et al., 2004; Lee et al., 2015). Therefore, it was not surprising to identify indoles in our assays. One of the metabolites found in higher concentrations in cultures that contained PCA and hematite together is structurally related to 5-hydroxyindole. Although a comparison to a commercial standard showed that this unidentified compound is not identical to 5-hydroxyindole, the similar MS2 fragmentation implies that it is related to this indole, and further investigation of its role in biological pathways in *M. barkeri* is needed.

### 4.3. *Methanosarcina barkeri* switch between methanogenesis and iron-reduction

Amorphous iron reduction by *M. barkeri* was shown to inhibit methanogenesis (Bond and Lovley, 2002; Van Bodegom et al., 2004; Sivan et al., 2016). The switch between these different processes was also observed in goethite (Liu et al., 2011b). Our study evaluated the archaea’s ability to switch between those two environmentally significant processes, with less reactive minerals than amorphous iron, in a non-H_2_ atmosphere. In the presence of PCA, *M. barkeri* switched between methanogenesis and iron reduction with all three iron-oxides tested in this study (Figure 4; Supplementary Figure S2). As the electron shuttle dramatically increased the iron-reduction and decreased the methanogenesis (Figure 3), it seems that PCA enables the archaea to favor iron-reduction over methanogenesis. Iron reduction is favored only when there is direct contact between the cells and the mineral, as shown nicely in Figure 7, hinting to the roles of methanophenazines, as mentioned above. When the conditions are less optimal for iron reduction, *M. barkeri* will carry out both methanogenesis and iron reduction.

The two processes (methanogenesis and iron reduction) thus display an inverse relationship (Supplementary Figure S2). Accordingly, while the magnitude of iron reduction was largest with amorphous iron, then magnetite, and eventually, with hematite, methane production showed the inverse trend: highest with hematite, then magnetite, and lowest with amorphous iron. The methane production in the hematite and magnetite-containing cultures without PCA, however, is not significantly different from the mineral free cultures. This indicates that iron does affect methanogenesis mainly in the presence of the electron shuttle. These observations also support the hypothesis that methanogens may respire iron oxides in nature, due to their methanophenazines.

Previous studies have shown that methanogens are able to utilize iron reduction for energy cell growth (Prakash et al., 2019; Yang and Lu, 2022). Our results also show that although *M. barkeri* did not thrive on iron reduction (Supplementary Figure S3), Fe^2+^ accumulation during the experiments suggested that the cultures are still active. The iron reduction rates were close to those of *Shewanella putrefaciens* (Supplementary Table S2). It should be noted that we cannot enterally rule out the possibility of cell lysis as a source for non-viable iron reduction, and the iron reduced in the control treatment may be attributed to this process. Nevertheless, the amount of iron reduced in our control experiments is less than 10% of the amount of iron reduced in live treatments.

Methanogenesis is a dominant reaction in anoxic environments, resulting in significant isotopic fractionation (Whiticar et al., 1986). The carbon isotopic composition of methane ( $\delta^{13}C_{{\mathrm{CH}}_{4}}$ ) excepted at the end of the second experiment also supports this observation of inhibition of methanogenesis by iron oxides. As a result of methanogenesis, the substrate pool in the methane-producing cultures becomes heavier along the experiment because the methanogens keep using more ^12^C than ^13^C. At some point, the archaea will exploit a significant portion of the light substrate present in the culture, leaving them to utilize the heavier substrate, resulting in heavier methane. The final $\delta^{13}C_{{\mathrm{CH}}_{4}}$ of methane-producing cultures was indeed ~10‰ higher than in the cultures where methanogenesis was inhibited almost completely.

### 4.4. The kinetics of iron reduction

In order to investigate the kinetics of the iron reduction reaction we introduced different concentrations of hematite, in the range of 0 to 10 mM (Figure 6A). Many parameters affect iron reduction rates, e.g., crystallinity, surface area (Roden, 2003), and the presence of electron shuttles (Lovley et al., 1996; Doong and Schink, 2002). The amount of reduced iron increased in almost all samples during the experiments. Interestingly, regardless of the concentration of iron in the samples, about 1% of hematite was reduced, indicating that the reaction rate is dependent on the iron concentration, resulting in a pseudo-first order reaction.

Another indication of the crucial role of the iron concentration in the reaction kinetics can be observed by the linear relationship of the added hematite to the produced Fe^2+^ (Figure 6B). This linear relationship indicates again that the iron concentration constitutes the barrier for this reaction and no other substrates, fitting first order reaction. The reduction rate coefficient was found to be 3.60 × 10^−3^ day^−1^. Although this reaction rate coefficient is not high, the reaction still occurs with hematite, the least reactive mineral examined in this experiment, and in a wide range of hematite concentrations, providing additional proof that iron reduction by methanogens potentially takes place in natural sediments. Interestingly, our observed iron reduction rate by the methanogens is only slightly lower than the known iron reducers *Shewanella putrefaciens* (and one order of magnitude less than the Geobacter genus; Supplementary Table S2). The fact that *M. barkeri* can reduce hematite with similar reaction rates as iron reducer establishes the potential role of this methanogen in iron reduction in deep sediment, and its ability to outcompete with bacterial iron reducers.

The substrate for the iron reduction is probably not methane, as ^13^C-labeled methane was not converted to enriched ^13^C-DIC. In addition, the slight methane concentration decrease in the living cultures without PCA cannot be related to AOM, because this decrease was also observed in cultures without iron oxides (Figure 4). The electron donor is hence probably from the inoculum. We flushed the vials very carefully, so a residual amount of hydrogen from the pre-culture is less likely, but we cannot rule it out. The reducing agent (Ti-Ct) is less likely based on our recent work (Shang et al., 2020), but also cannot be ruled out. The most probable electron donor is the yeast extract itself (Supplementary methods S1). However, the exact organics in the yeast are less relevant to this study.

### 4.5. Environmental implications

Deep sediments have complex microbial redox couplings that affect global biogeochemical cycles, such as carbon, and as a result, it affects all living species on Earth. Methane, a significant greenhouse gas, is consumed and produced in those sediments and can be released into the atmosphere. Iron reduction by lowly reactive iron minerals in the methanogenic zone was previously demonstrated (Gault et al., 2011; Egger et al., 2017; Vigderovich et al., 2019; Amiel et al., 2020) but the involved dominant microbes have not been identified yet. It has been suggested that archaeal methanogens may play a role in this reduction, based on geochemical and microbial studies in natural sediments (Elul et al., 2021). Here, we indeed established that the naturally abundant archaeon methanogen *M. barkeri* might actually switch its metabolic pathway from methanogenesis to iron reduction, with abundant and low-reactivity minerals such as magnetite and hematite, and in the absence of hydrogen. Further proof that the metabolic switch indeed occurs in nature is still required, but the fact that this process readily takes place in pure cultures without H_2_ addition can shed light on the advantage of iron reduction for methanogens such as *M. barkeri* in nature. The thermodynamics of iron reduction of magnetite and hematite are similar to those related to methane production, so favoring this specific reaction is probably due to kinetic limitations and not thermodynamics. We also showed that hematite reduction by *M. barkeri* is a pseudo-first order reaction, indicating that in deep sediments, where iron minerals are abundant, *M. barkeri* could serve as an iron reducer, depending only on the available Fe(III) concentration. *M. barkeri* can thus switch from methanogenesis to reduce abundant iron oxides (Bar-Or et al., 2017; Vigderovich et al., 2019; Amiel et al., 2020) to survive in this environment. Further studies are needed to establish and characterize the switch between iron reduction and methane production by methanogens in nature. A better understanding of the processes in deep sediments will provide important insights into the biogeochemical cycles of iron and the greenhouse gas methane.

## 5. Conclusion

We studied the potential of the archaeal methanogen *M. barkeri* to reduce iron in close to natural conditions. We showed that *M. barkeri* could reduce not only high reactive iron-oxides such as amorphous iron but also low-reactivity minerals such as magnetite and hematite. Moreover, we showed that electron shuttles could promote these processes, but direct contact with the mineral and the cells is needed, pointing to the important role of methanophenazines in the advantage of methanogens over iron reducing bacteria in reducing the iron in deep methanogenic sediments. Hematite reduction by *M. barkeri* was found to be a pseudo-first order reaction, indicating that in deep sediments, where iron minerals are abundant, *M. barkeri* could serve as an iron reducer, depending only on the available Fe(III) concentration. If indeed this methanogen is responsible for the detected elevation of Fe^2+^ in the bottom of anoxic sediments, it could reveal another unknown process involving the potent greenhouse gas methane. Further investigation should take place to evaluate the extent of this biological process in the natural environment in order to assess its impact on the carbon cycle.

## Data availability statement

The original contributions presented in the study are included in the article/Supplementary material, further inquiries can be directed to the corresponding authors.

## Author contributions

OS, ZT, and EE-R designed the experiments. ZT and EE-R performed them and analysed the data together with all authors. The manuscript was written through contributions of all authors. All authors contributed to the article and approved the submitted version.

## Funding

This project was funded by a European Research Council Consolidator Grant (818450) to OS, and by the Israel Science Foundation Personal Grant (1485/20 to MMM and 1573/22 to OS).

## Conflict of interest

The authors declare that the research was conducted in the absence of any commercial or financial relationships that could be construed as a potential conflict of interest.

## Publisher’s note

All claims expressed in this article are solely those of the authors and do not necessarily represent those of their affiliated organizations, or those of the publisher, the editors and the reviewers. Any product that may be evaluated in this article, or claim that may be made by its manufacturer, is not guaranteed or endorsed by the publisher.
