# Supplementary material for: The reduction of environmentally abundant iron oxides by the methanogen Methanosarcina barkeri
Source: Front Microbiol. 2023 Jul 20;14:1197299. doi: 10.3389/fmicb.2023.1197299 (PMC10399698; doi:10.3389/fmicb.2023.1197299)
Supplement: Supplementary file 1 [file Data_Sheet_1.docx]

Supplementary Material

# Supplementary Data

**1.1 Methods S1** Methanogens medium protocol.

The methanogens medium was prepared as describe by ref (Boone et al., 1989) with some modification:

4.0 g NaOH

1.0 g Yeast extract

1.0 g Trypticase peptones

0.5 g Mercaptoethanesulfonic acid

0.4 g K_2_HPO_4_

1.0 g NH_4_Cl

1.0 g MgCl_2_∙6H_2_O

0.4 g CaCl_2_∙2H_2_O

5.0 mg NaEDTA∙2H_2_O

1.5 mg CoCl_2_∙6H_2_O

1.0 mg MnCl_2_∙4H_2_O

1.0 mg FeSO_4_∙7H_2_O

1.0 mg ZnCl_2_

0.4 mg AlCl_3_∙6H_2_O

0.3 mg Na_2_WO_4_∙2H_2_O

0.2 mg CuCl_2_∙2H_2_O

0.2 mg NiSO_4_∙6H_2_O

0.1 mg H_2_SeO_3_

0.1 mg BH_3_O_3_

0.1 mg NaMoO_4_∙2H_2_O

Deionized H_2_O were added up to volume of 1 L. Anaerobic atmosphere of 80:20 H_2_:CO_2_ was created and the pH was adjusted to 7 and autoclaved to sterilize.

Additional solutions were prepared as follow:

10 mL Vitamin Solution (DSMZ 141):

2.0 mg Biotin

2.0 mg Folic acid

10.0 mg Pyridoxine-HCl

5.0 mg Thiamine-HCl∙2H_2_O

0.5 g Riboflavin

5.0 mg Nicotinic acid

5.0 mg D-Ca-pantothenate

0.1 mg Vitamin B_12_

5.0 mg p-Aminobenzoic acid

5.0 mg Lipoic acid

Deionized H_2_O were added up to volume of 1 L, sterilized by 0.22 µm filter under anaerobic conditions.

Reducing agent:

Titanium (III) citrate stock solution (concentration of 256 mM):

3.75 mL 20% Ti(III)Cl_3_ (anaerobic)

50 mL 0.2 M Na-Citrate (anaerobic)

Neutralized with saturated sodium carbonate to pH 7, autoclaved to sterilize.

To each culture 1% of vitamins solution and titanium (III) citrate to final concentration of 2.5 mM were added anaerobically.

# Supplementary Figures and Tables

## Supplementary Tables

| Bottles | Hem.  (mM) | Mag.  (mM) | AmFeIII  (mM) | PCA (mM) | AQDS (mM) | ^13^CH_4_ | δ^13^C DIC | δ^13^C CH_4_ | 16S rRNA | Auto-claved | Head-space |
| --- | --- | --- | --- | --- | --- | --- | --- | --- | --- | --- | --- |
| **Exp I** |  | | | | | | | | | | |
| A 1,2 | 10 | - | - | 0.1 | - | + | + | - | - | - | N_2_:CO_2_  90:10 |
| B 1,2 | 10 | - | - | - | 0.1 | + | + | - | - | - |  |
| C 1,2 | 10 | - | - | - | - | + | + | - | - | - |  |
| D 1,2 | - | - | - | - | 0.1 | - | - | - | - | X |  |
| **Exp II** |  | | | | | | | | | | |
| A 1,2 | - | - | - | - | - | - | + | + | - | - | N_2_:CO_2_  90:10 |
| B 1,2 | - | - | - | 0.1 | - | - | + | + | - | - |  |
| C 1,2,3 | - | - | - | 0.1 | - | - | - | - | - | X |  |
| D 1,2,3 | 10 | - | - | - | - | - | + | + | - | - |  |
| E 1,2,3 | 10 | - | - | 0.1 | - | - | + | + | - | - |  |
| F 1,2,3 | 10 | - | - | 0.1 | - | - | - | - | - | X |  |
| G 1,2,3 | - | 10 | - | - | - | - | + | + | - | - |  |
| H 1,2,3 | - | 10 | - | 0.1 | - | - | + | + | - | - |  |
| I 1,2,3 | - | 10 | - | 0.1 | - | - | - | - | - | X |  |
| J 1,2,3 | - | - | 10 | - | - | - | + | + | - | - |  |
| K 1,2,3 | - | - | 10 | 0.1 | - | - | + | + | - | - |  |
| L 1,2,3 | - | - | 10 | 0.1 | - | - | - | - | - | X |  |
| **Exp III** |  | | | | | | | | | | |
| A 1,2 | - | - | - | - | - | - | + | - | - | X | N_2_:CO_2_  80:20 |
| B 1, 2, 3 | - | - | - | - | - | - | + | - | - | - |  |
| C 1, 2, 3 | 10 | - | - | - | - | - | + | - | - | - |  |
| D 1, 2, 3 | - | - | - | 0.1 | - | - | + | - | - | - |  |
| E 1, 2, 3 | 10 | - | - | 0.1 | - | - | + | - | - | - |  |
| **Exp IV** | | | | | | | | | | | |
| A 1,2 | - | - | - | - | - | - | - | - | +. | - | N_2_:CO_2_  80:20 |
| B 1,2 | 0.1, 0.2 | - | - | - | - | - | - | - | + | - |  |
| C 1,2 | 1.0 | - | - | - | - | - | - | - | + | - |  |
| D 1,2 | 10 | - | - | - | - | - | - | - | + | - |  |
| E 1 | - | - | - | - | - | - | - | - | + | - | H_2_:CO_2_  80:20 |
| **Exp V** |  | | | | | | | | | | |
| A 1,2, 3 | - | - | - | - | - | - | + | - | - | X | N2 only |
| B 1, 2 | - | - | - | - | - | - | + | - | - | - |  |
| C 1, 2, 3 | 10, | - | - | - | - | - | + | - | - | - |  |
| D 1, 2, 3 | 10, in cryo vial * | - | - | - | - | - | + | - | - | - |  |

*Direct contact of *M. barkeri* and Hematite was prevented.

**Supplementary Table 1.** Experimental design: A,B,C… denotes treatments. 1,2,3 denotes repetitions (duplicates/ triplicates) in the same experiment. Abbreviations and volumes for final concentrations in the bottles: Hematite (Hem., 1 mL), Magnetite (Mag., 1 mL), amorphous iron (Fe(III)OH_3_, 1 mL), PCA (1 mL), AQDS (1 mL), ^13^CH_4_ (2 mL).

| Source | Hematite reduction rate [µM/day] | CFU [cells/mL] | Beginning hematite concentration | Organism |
| --- | --- | --- | --- | --- |
| This study | 36 | 1.5 ∙10^8 a^ | 10 mM | *M. barkeri* |
| (Royer et al., 2004) | 53 | 10^8 b^ | 25 mM | *Shewanella putrefaciens* |
| (Weihe et al., 2019) | 192 | 2∙10^8^ | 1.27 mM | *Shewanella oneidensis* |
| (Bosch et al., 2010) | 298 | 2.1∙10^8^ | 9.6 mM | *Geobacter sulfurreducens* |

^a^ Average of two cultures.

^b^ 2% hydrogen were presence in the experiment.

**Supplementary Table 2.** Hematite reduction rates by different organisms.

## Supplementary Figures


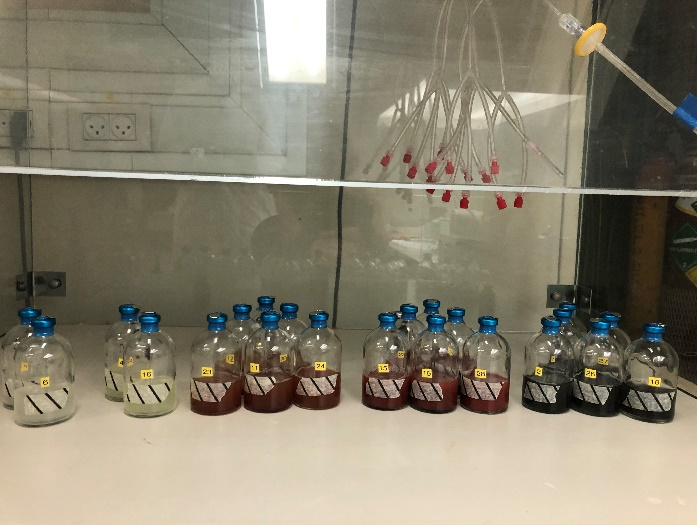

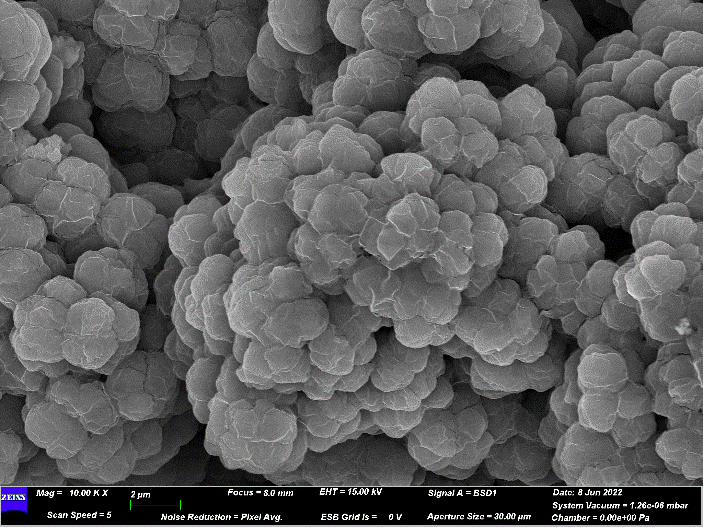

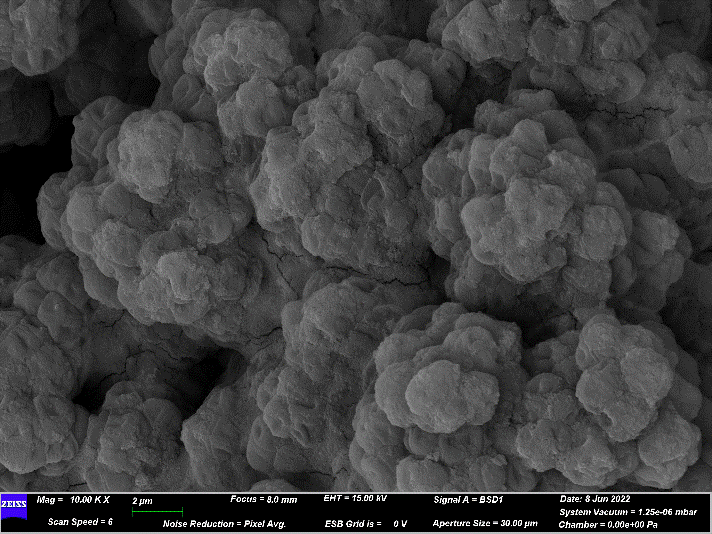


**A** mineral free

**B** amorphous iron

*M.barkeri*, measured cell diameter 2µm

*M.barkeri*, coated


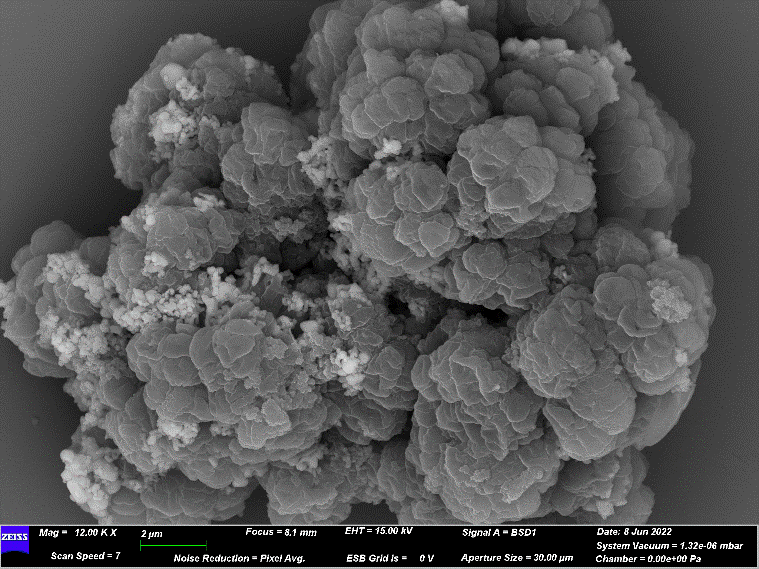


**C** hematite

Secondary iron bearing minerals oxide precipitation between *M. barkeri* cells*.*

Supplementary Figure 1. Experiment II treatments bottles on the first day of the experiment (upper part), and SEM images in the end of the experiment (lower part). SEM images of the of M.barkeri culture with (A) mineral free, (B) Amorphus iron and (C) Hematite. Cells fixed as described by (Wang et al., 2020) and scanned with Zeiss Gemini (scale 2µM, 15KV).

**Supplementary Figure 2.** Methanogenesis rates vs Fe oxides reduction rates (Hem=Hematite, Mag=Magnetite, Amr= poorly crystalline, PCA= poorly crystalline ferrihydrite). Each data point represents triplicate or duplicate treatments with Fe oxide concentration of 10 mM.

**Supplementary Figure 3.** Experiment III calculated cell count is based on 16S rRNA qPCR analyses at the end of experiment (day 11), assuming 3 gene copies of 16S rRNA in Archea. Treatments of varied hematite concentration (0, 0.1, 0.2, 1, 10 mM) grown under N_2_:CO_2_ (80:20) atmosphere. Control as culture under H_2_ measured at the beginning of experiment.

**References**

Boone, D. R., Johnson, R. L., and Liu, Y. (1989). Diffusion of the Interspecies Electron Carriers H2 and Formate in Methanogenic Ecosystems and Its Implications in the Measurement of Km for H2 or Formate Uptake. *Appl. Environ. Microbiol.* 55, 1735–1741. doi:10.1128/AEM.55.7.1735-1741.1989.

Bosch, J., Heister, K., Hofmann, T., and Meckenstock, R. U. (2010). Nanosized iron oxide colloids strongly enhance microbial iron reduction. *Appl. Environ. Microbiol.* 76, 184–189. doi:10.1128/AEM.00417-09.

Royer, R. A., Dempsey, B. A., Jeon, B. H., and Burgos, W. D. (2004). Inhibition of Biological Reductive Dissolution of Hematite by Ferrous Iron. *Environ. Sci. Technol.* 38, 187–193. doi:10.1021/ES026466U.

Wang, H., Byrne, J. M., Liu, P., Liu, J., Dong, X., and Lu, Y. (2020). Redox cycling of Fe(II) and Fe(III) in magnetite accelerates aceticlastic methanogenesis by Methanosarcina mazei. *Environ. Microbiol. Rep.* 12, 97–109. doi:10.1111/1758-2229.12819.

Weihe, S. H. C., Mangayayam, M., Sand, K. K., and Tobler, D. J. (2019). Hematite Crystallization in the Presence of Organic Matter: Impact on Crystal Properties and Bacterial Dissolution. *ACS Earth Sp. Chem.* 3, 510–518. doi:10.1021/ACSEARTHSPACECHEM.8B00166.
